# Antecedent Treatment with Different Antibiotic Agents as a Risk Factor for Vancomycin-Resistant *Enterococcus*

**DOI:** 10.3201/eid0808.010418

**Published:** 2002-08

**Authors:** Yehuda Carmeli, George M. Eliopoulos, Matthew H. Samore

**Affiliations:** * Beth Israel Deaconess Medical Center, Boston, Massachusetts, USA; † Harvard Medical School, Boston, Massachusetts, USA

**Keywords:** vancomycin, Enterococcus, resistance, risk factor

## Abstract

We conducted a matched case-control study to compare the effect of antecedent treatment with various antibiotics on subsequent isolation of vancomycin-resistant *Enterococcus* (VRE); 880 in-patients; 233 VRE cases, and 647 matched controls were included. After being matched for hospital location, calendar time, and duration of hospitalization, the following variables predicted VRE positivity: main admitting diagnosis; a coexisting condition (e.g., diabetes mellitus, organ transplant, or hepatobiliary disease); and infection or colonization with methicillin-resistant *Staphylococcus aureus* or *Clostridium difficile* within the past year (independent of vancomycin treatment). After controlling for these variables, we examined the effect of various antibiotics. Intravenous treatment with third-generation cephalosporins, metronidazole, and fluoroquinolones was positively associated with VRE. In our institution, when we adjusted the data for temporo-spatial factors, patient characteristics, and hospital events, treatment with third-generation cephalosporins, metronidazole, and fluoroquinolones was identified as a risk factor for VRE. Vancomycin was not a risk factor for isolation of VRE.

First isolated in the late 1980s (1,2), vancomycin-resistant enterococci (VRE) have rapidly become established as important nosocomial pathogens in the United States. In some hospitals, VRE are responsible for >20% of enterococcal infections (3).

 Given the complex genetic machinery required to confer vancomycin resistance, de novo emergence of resistance is unlikely in an individual patient (4). Thus, newly detected VRE may represent either acquisition of resistant organisms (or genes) or expansion of preexisting but undetected populations of VRE with which the patient is colonized (5). The likelihood of nosocomial VRE may vary with time and space, according to the endemicity of VRE in a specific location (i.e., colonization pressure) and to the duration of hospitalization (i.e., time at risk) (6,7). Indeed, initially most VRE isolates were recovered from patients in intensive-care units (ICUs); later VRE became more prevalent in patients on other wards (3). Certain coexisting conditions, e.g., malignancies, organ transplants, and chronic renal failure, were found to be associated with increased risk for VRE, as were exposure to contaminated equipment and proximity to a VRE carrier (8–16).

The effect of antecedent treatment with various antibiotic agents as a risk factor for nosocomial VRE has been explored in numerous studies, with conflicting results. Antimicrobial agents are believed to predispose to nosocomial VRE largely through effects on competing gastrointestinal microflora. Epidemiologic studies have identified therapy with vancomycin as a risk factor for VRE infection or colonization (8–19). A few studies have demonstrated an association between VRE and other antibiotic agents, including cephalosporins, quinolones, and metronidazole (12,14,18,19). However, no published study has directly compared multiple antibiotic agents while controlling for confounding.

Recently, we systematically reviewed published studies and provided evidence that questioned the relationship between vancomycin use and individual risk for nosocomial VRE colonization and infection (20). We suggested that the reported association might result from confounding as a result of selection of an inappropriate control group, lack of control for differences between cases and controls in duration of hospital stay, and publication bias. To conduct a study that examines multiple antibiotic agents simultaneously while controlling for confounding, a large number of VRE cases and controls are needed. Ideally designed, a study should be conducted in which serial cultures are collected prospectively to document the timing of change in patient status from VRE negative to VRE positive. However, such a study will be expensive and labor intensive. Only a few studies have been performed in which serial cultures were taken; these were conducted at high-incidence units and their small sample size made it difficult to control for multiple confounding (7,19,21). Thus, a retrospective study in which patients are included on the basis of clinical cultures was the only practical option.

Using this approach, we conducted a matched case-control study comparing the effect on VRE isolation of antecedent treatment with various antibiotics while controlling for temporo-spatial factors such as length of stay, hospital location, and calendar time, as well as patient characteristics.

## Methods

The Beth Israel Deaconess Medical Center–West Campus is a 320-bed urban tertiary-care teaching hospital in Boston, Massachusetts. It has 24 ICU beds and approximately 12,000 patient admissions each year. The institutional antibiotic policy requires approval by an infectious disease consultant for the use of third-generation cephalosporins (other than ceftriaxone), antipseudomonal agents, and vancomycin (for more than one dose).

 Data were collected from administrative, pharmacy, clinical, and laboratory computerized databases by using a relational database management system (Access, Microsoft Corp., Redmond, WA). The databases and methods of data collection have been described (22).

 Enterococci were identified from clinical specimens submitted to the microbiology laboratory by using the Gram-Positive Identification Panel (Dade Behring Inc., Deerfield, IL). Enterococci were screened for vancomycin resistance by plating on brain heart infusion agar with 6 µg/mL vancomycin. Vancomycin resistance was confirmed by formal MIC testing with the microdilution broth method (MicroScan, Dade International Inc.). Isolates with vancomycin MICs ≥8 µg/mL were classified as VRE.

### Definitions and Study Design

The study was designed as a matched case-control study. All inpatients from whom VRE were first isolated from a clinical culture (either infected or colonized patients) in our hospital from October 1, 1993, through December 31, 1997, were enrolled as cases. Patients transferred from another institution and known to be VRE positive at that time were not included in this study. Patients and controls were matched on the basis of three variables: hospital ward, calendar time (within 7 days), and duration of hospital stay at the time of matching (up to 3 days’ difference if no exact match was available). Up to three appropriately matched control-patients who were not VRE positive (i.e., patient was cultured and no VRE were isolated or the patient was never cultured) were randomly selected for each case. A list of all possible controls was created. Each was assigned a random number, and the three highest random numbers were chosen (without replacement). We looked for risk factors by examining demographics, admitting diagnosis, coexisting conditions (based on ICD-9 codes and electronic records), transfer from another institution, admission to an ICU and number of days in ICU, major surgical procedure, and infection with *Clostridium difficile* or methicillin-resistant *Staphylococcus aureus* (MRSA). After controlling for confounding by these variables, we compared, in detail, antecedent treatments with different antibiotic agents.

### Statistical Analysis

Statistics were run on Stata (Stata Corp., College Station, TX) software. A matched (conditional) logistic regression model was used. All variables other than antibiotic exposures were candidates for the model and were selected in a stepwise manner with an entry criterion of p<0.2 and a criterion to stay in the model of p<0.05. Variables that were not retained in the model by this procedure were then tested for confounding by adding them one at a time to the model and examining their effects on the β-coefficients. Variables that caused substantial confounding (change in β-coefficient of >10%) were included in the final model. After constructing the explanatory model, we examined the effect of treatment with each antibiotic by adding them to the model. The effects of antibiotic treatment were also examined by including them in the model and excluding possible collinear variables that were part of the explanatory model (e.g., vancomycin and infection or colonization with MRSA). In addition to examining statistical significance and confounding, we evaluated effect modification between variables by testing appropriate interaction terms for statistical significance. All statistical tests were two-tailed. A value of p<0.05 was considered significant.

## Results

 During the 51-month study period, the incidence of VRE increased from 34 to 88 cases per 10,000 admissions. VRE were isolated in clinical cultures from 251 patients who fulfilled the study criteria (first isolation of VRE while hospitalized in our institution). The 251 diagnostic cultures were sent from 30 different nursing units. Twenty-eight percent of the case-patients were diagnosed during an ICU stay. No appropriate control patient could be matched for 18 cases. Thus, the study included 880 patients—233 cases and 647 matched control patients. The average age was 62 years (range 17–105), and 46% of the patients were female. Patients were hospitalized for an average of 8.1 days before entry into the study. The likelihood of being cultured (between admission and 2 days before matching) for cases and controls had similar distribution of the likelihood of being cultured (median 0, 0; 75th percentile 0, 1; and 90th percentile 21, 24 cultures for controls and cases, respectively).

The patients’ characteristics with the unadjusted associated relative risks (odds ratios [OR]) for nosocomial VRE are shown in Table 1. Univariate matched analysis showed that case-patients were more likely than controls to be hospitalized for gastrointestinal and infectious conditions and less likely to be admitted for a cardiovascular condition. Case-patients were also more likely than controls to be solid organ transplant recipients and to have one of the following coexisting conditions: diabetes mellitus, renal disease, or hepatobiliary disease. Case-patients had higher chronic coexisting condition (Charlson) scores than controls and were less likely to have had major surgery during the index admission. Case-patients were also more likely than controls to have been infected (or colonized) within the past year with MRSA or *C. difficile*.

**Table 1:** Patient characteristics and matched univariate analysis for isolation of nosocomial vancomycin-resistant enterococci

Variable	Cases (%) (233)	Control (%) (647)	Odds ratio	p value
Age^a^	61.7	62.3	.999	0.76
Gender (female)	114 (49)	292 (45)	1.2	0.34
Main admitting diagnosis				
Orthopedic condition	5 (6.4)	53 (8.2)	R	R
Cardiovascular condition	49 (21)	236 (36)	0.37	<0.001
Endocrine disorder	6 (2.6)	13 (2)	1.3	0.6
Gastrointestinal disorder	77 (33)	160 (25)	1.7	0.005
Genitourinary disorder	18 (7.7)	35 (5.4)	1.4	0.21
Infectious disease	26 (11)	19 (3)	3.7	<0.001
Hematologic disease	5 (2.1)	12 (1.9)	0.83	0.7
Neurologic disease	18 (7.7)	69 (10.7)	0.72	0.3
Pulmonary disease	19 (8.1)	49 (7.6)	1.1	0.8
Coexisting conditions				
Cardiovascular disease	160 (69)	460 (71)	0.85	0.36
Lung disease	32 (14)	88 (14)	1	0.95
Diabetes mellitus	127 (55)	262 (40)	1.9	<0.001
Organ transplant recipient	39 (17)	45 (7)	2.9	<0.001
Renal disease	57 (24)	103 (16)	1.6	0.013
Cancer	25 (11)	93 (14)	.71	0.18
AIDS	2 (1)	8 (1)	.75	0.7
Hepatobiliary disease	65 (28)	97 (15)	2.5	<0.001
Charlson comorbidity score^a^	3.2	2.7	1.114	0.003
Transfer from an institution	84 (36)	243 (37)	0.9	0.56
Surgery	67 (29)	211 (33)	0.55	0.01
Admission to ICU	65 (28)	169 (26)	.78	0.38
MRSA				
During current admission	18 (8)	16 (2.5)	3.9	0.001
In past year	28 (12)	26 (4)	3.5	<0.001
*Clostridium difficile*				
During current admission	5 (2.1)	10 (1.5)	1.3	0.59
In past year	17 (7.3)	20 (3.1)	2.6	0.006

^a^Continuous variable. R, reference group; MRSA, methicillin-resistant *Staphylococcus aureus*.

 We developed a multivariate model to explain the likelihood of being VRE positive (Table 2). After being matched for hospital location, calendar time, and duration of hospitalization, the following variables predicted being VRE positive: 1) main admitting diagnosis; 2) coexisting conditions of diabetes mellitus, organ transplant, or hepatobiliary disease; and 3) infection or colonization with MRSA or *C. difficile* within the past year. In the model adjusting for these variables, we examined the effect of being treated with each antibiotic (the adjusted effect). The unadjusted and adjusted effects of antecedent treatment with each agent are summarized in Table 3. Univariate (unadjusted) matched analysis disclosed that case-patients were more likely to have been treated with intravenous penicillins, third-generation cephalosporins, vancomycin, metronidazole, and quinolones (ciprofloxacin and ofloxacin). After we controlled for confounding, only intravenous treatment with cephalosporins, in particular third-generation cephalosporins, and with metronidazole was positively associated with VRE. Few patients were treated with oral antibiotics (<6%), and no significant association was found between enteral vancomycin (OR 1.2, p=0.83), metronidazole (OR 1.5, p=0.29), or clindamycin (OR 1.0, p=0.9) and VRE.

**Table 2:** Multivariable explanatory model for having vancomycin-resistant enterococci-positive case

Variable	Odds ratio (95% CI)	p value
Main admitting disorder	0.44 (0.28 to 0.68)	<0.001
Cardiovascular	2.9 (1.5 to 5.7)	0.002
Infectious		
Coexisting conditions		
Diabetes mellitus	2.1 (1.5 to 3.1)	<0.001
Transplant recipient	2.6 (1.6 to 4.5)	<0.001
Hepatobiliary disease	2.9 (1.8 to 4.6)	<0.001
MRSA (in past yr) *Clostridium difficile* (in past yr)	3.5 (1.8 to 6.9) 2.0 (0.97 to 4.3)	<0.001 0.06

CI, confidence interval; MRSA, methicillin-resistant *Staphylococcus aureus*.

**Table 3:** The effect of antibiotic treatment as risk factor for vancomycin-resistant enterococci

	Cases (%)	Control (%)	Unadjusted effect		Adjusted for explanatory model^a^		Adjusted for model and other antibiotics^a^	
Antibiotic agent	(233)	(647)	OR	p value	OR (95% CI)	p value	OR (95% CI)	p value
Penicillins	67 (29)	134 (21)	1.5	0.04	.99 (.63 to 1.6)	0.97	1.0 (.64 to 1.7)	0.86
β-lactam-inhibitor combination	49 (21)	98 (15)	1.5	0.07	.94 (.6 to 1.5)	0.78		
Cephalosporins	104 (45)	248 (38)	1.2	0.28	1.5 (1.0 to 2.4)	0.048		
Third generation	69 (30)	97 (15)	2.6	<0.001	2.8 (1.7 to 4.5)	<0.001	2.8 (1.7 to 4.8)	<0.001
Vancomycin (p.o.)	4 (1.7)	7 (1.1)	1.2	083	1.0 (.25 to 4.2)	0.96		
Vancomycin (i.v.)	67 (29)	121 (19)	1.7	0.016	1.4 (.89 to 2.3)	0.19	.99 (.57 to 1.7)	0.98
Metronidazole (p.o.)	13 (5.6)	23 (3.6)	1.5	0.29	1.0 (.42 to 2.5)	0.97		
Metronidazole (i.v.)	47 (20)	57 (9)	2.5	<0.001	2.3 (1.3 to 3.9)	0.003	2.1 (1.2 to 3.7)	0.008
Clindamycin	20 (8.6)	51 (7.9)	1	0.9	1.5 (.76 to 2.8)	0.26	1.1 (.55 to 2.3)	0.76
Quinolone^b^	48 (21)	68 (11)	2	0.005	1.6 (.94 to 2.6)	0.086	1.5 (.85 to 2.6)^b^	0.17^b^
Imipenem	19 (8.2)	27 (4.2)	1.7	0.12	1.3 (.61 to 2.9)	0.47	1.2 (.52 to 2.8)	0.66

^a^Adjusted for the explanatory model detailed in Table 2. ^b^ When included as a continuous variable (number of days of treatment with quinolone) OR=1.03, p=0.05. OR, odds ratio; p.o., orally; i.v., intravenously.

We examined the data to determine whether colinearity of vancomycin with MRSA or *C. difficile* infection explained the lack of significance of parenteral vancomycin in the adjusted analysis. A model that included vancomycin but excluded MRSA or *C. difficile* infection was constructed. In this model as well, treatment with vancomycin was not associated with VRE positivity (OR 1.3; p=0.8).

 We also examined the effect of duration of treatment with each of the agents studied. The duration of treatment with vancomycin (OR 0.99; p=0.66), metronidazole (OR 1.03; p=0.18), and third-generation cephalosporins (OR 1.01, p=0.66) was not associated with VRE. In contrast, longer treatment with quinolones was associated with VRE both in the unadjusted analysis (OR 1.03; p=0.03) and in the multivariate model (OR 1.03; p=0.05).

 In our final model (Table 3), the antibiotics included were third-generation cephalosporins (OR 2.9; p<0.001), intravenous metronidazole (OR 2.0; p=0.012), and long-term use of fluoroquinolones (OR 1.034; p=0.027).

## Discussion

 VRE is a major emerging pathogen that has spread rapidly since these organisms were first detected approximately a decade ago (23). Antibiotics, particularly vancomycin, have been ascribed a crucial role in the dissemination of VRE; yet, many publications addressing this subject had small sample sizes or control groups, focused on a limited number of antimicrobial agents, or did not completely control for confounding factors. Thus, the true relationship between vancomycin and VRE and the relative importance of antimicrobial agents other than vancomycin have remained unclear.

In this study, the largest reported to date on VRE, we systematically compared the major classes of antibiotics used in the hospital setting for their association with VRE infection. The effects of duration of treatment and route of administration (i.e., oral and parenteral) were also examined. We controlled for temporo-spatial factors, correlates of transmission, and duration of risk by matching case-patients and controls for hospital location, calendar time, and duration of hospital stay until diagnosis. We considered length of stay to be particularly important because it represents the duration of the at-risk period for both exposure to antibiotics and acquisition of VRE and, in addition, is a correlate of severity of illness. Multivariable models were used to address other potential confounding factors such as surgical procedures, coexisting conditions, and reason for hospitalization.

Our major findings were 1) vancomycin was not associated with VRE positivity, a finding consistent with the results of the meta-analysis on this subject (20); 2) third-generation cephalosporins and parenteral metronidazole were highly significant independent risk factors for VRE; and 3) only fluoroquinolones exhibited a statistically significant linear relationship between intensity (duration) of exposure and risk for VRE. In contrast, for metronidazole and third-generation cephalosporins, a threshold-type (all or none) effect was observed. The risk for VRE in patients treated with these agents was increased regardless of duration of therapy.

The small effect of parenteral vancomycin in the unadjusted analysis was completely erased after the data were controlled for confounding by patient characteristics and treatment with other antibiotics, mainly treatment with third-generation cephalosporins and metronidazole. Thus, individual patients who received vancomycin did not appear to be at any increased risk for VRE infection. We believe that the lack of effect of vancomycin on VRE found in this study, a finding that contradicts the results of many earlier studies, relates to our compliance with adequate epidemiologic principles in study design and analysis. These principles include controlling for length of stay, choosing the control group from the source population, matching for endemicity by matching on time and location, and adjusting for other antibiotic exposures. Most early studies that identified vancomycin as a strong risk factor for VRE failed to account for these principles (24,25).

 These data do not dispute the role of glycopeptide use in promoting the emergence of glycopeptide resistance; this role may be related to glycopeptides’ effect on the possibility of a positive patient’s becoming a transmitter, rather than on increasing the risk of the susceptible person’s becoming colonized (26). Indeed, our study was aimed at the individual level and not at the group level. A recent study performed at the group level demonstrated that ICUs in which vancomycin is heavily used have higher rates of VRE (27). We believe that the discordant results between individual level and group level analysis (28) and the effect of glycopeptides on the possibility of transmission among the already colonized patients deserve further study. The results of our analysis, as well as results of other studies (6,7,20), call into question whether restricting vancomycin will lower VRE incidence.

The effect of third-generation cephalosporins on risk for VRE is likely due to their activity against nonenterococcal aerobic enteric flora, leading to decrease in resistance colonization, allowing colonization with VRE. Similarly, suppression of gastrointestinal anaerobic flora is the presumed mechanism for the association between metronidazole and VRE. This activity and suppression do not explain the lack of effect of other agents with similar or even broader spectra of activity such as clindamycin, β-lactamase–inhibitor combinations, and imipenem. Other researchers have suggested that the combination of enteric concentration of the antimicrobial agent and its spectrum of activity against competing microflora determines its likelihood to be a risk factor (Rice LB, unpub. data). In our study, the positive association between duration of quinolone treatment and VRE had borderline statistical significance and a small increased risk per day of treatment. This observation, which requires further validation, may have important clinical importance for patients treated for long durations.

We also found that patients who were VRE positive were more likely to have been infected or colonized with MRSA or *C. difficile* in the past year. This association has been previously described (29) and, in our data, was independent of vancomycin treatment. This relationship likely reflects shared mechanisms of acquisition for these nosocomial pathogens and a common association with severity of illness.

Our study has certain limitations. We assumed that time of VRE positivity was similar to time of acquisition for cases. This assumption is likely incorrect but is the best possible estimate in this type of study. Studies based on serial surveillance cultures may yield a more accurate estimate of time of acquisition but cannot reach an adequate sample size to perform statistical analysis controlling for confounding. If we had performed serial cultures twice a week on all our source population, we would have processed >100,000 surveillance cultures. Indeed, almost all previous studies on this subject had a similar assumption. Control patients were representative of the hospital-based population but were not screened to exclude undetected VRE colonization. However, it is unlikely that misclassification bias could simultaneously account for the substantial effect observed with certain antibiotics and lack of effect observed with others. Moreover, the results of a meta-analysis also suggest that the magnitude of association between vancomycin treatment and VRE was independent of the method of VRE detection, i.e., clinical or surveillance cultures (20). Another caveat is that the results of this study apply to individual risk for VRE. Antibiotics may have differential effects on the quantity of VRE excreted from already colonized persons, as suggested both by animal models and human data (30–32). Thus, the effects of antibiotics on ecologic risk, e.g., transmission of VRE to other patients, may differ from their effects on individual risk (28). Finally, the power of this study to examine the effects of oral antibiotics was limited because of the small number of patients treated with these agents. Along the same lines, because of their limited use, these agents are unlikely to play a major role in the epidemiology of VRE within hospitals.

We conclude that patients treated with third-generation cephalosporins, metronidazole, or quinolones for an extended duration appear to be at significantly higher risk for VRE. Antecedent treatment with vancomycin is not a risk factor for VRE infection or colonization. Further studies to examine the routes of transmission of VRE and the ecologic role of antibiotics are needed.

## References

[1] Leclercq R, Derlot E, Duval J, Courvalin P. Plasmid-mediated resistance to vancomycin and teicoplanin in *Enterococcus faecium.* N Engl J Med. 1988;319:157–61. 2968517 10.1056/NEJM198807213190307

[2] Uttley AHC, Collins CH, Naidoo J, George RC. Vancomycin-resistant enterococci. Lancet. 1988;1:57–8. 10.1016/S0140-6736(88)91037-9 2891921

[3] Centers for Disease Control and Prevention. National nosocomial infections surveillance (NNIS) report, data summary from October 1986-April 1996, issued May 1996. A report from the National Nosocomial Infections Surveillance (NNIS) system. Am J Infect Control. 1996;24:380–8. 10.1016/S0196-6553(96)90026-7 8902113

[4] Evers S, Casadwall B, Charles M, Dutka-Malen S, Galimand M, Courvalin P. Evolution of structure and substrate specificity in D-alanine:D-alanine ligases and related enzymes. J Mol Evol. 1996;42:706–12. 10.1007/BF02338803 8662022

[5] Van der Auwera R, Pensart N, Korten V, Murray BE, Leclercq R. Influence of oral glycopeptides on the fecal flora of human volunteers: selection of highly glycopeptide-resistant enterococci. J Infect Dis. 1996;173:1129–6. 8627064 10.1093/infdis/173.5.1129

[6] Austin DJ, Bonten MJ, Weinstein RA, Slaughter S, Anderson RM. Vancomycin-resistant enterococci in intensive-care hospital settings: transmission dynamics, persistence, and the impact of infection control programs. Proc Natl Acad Sci U S A. 1999;96:6908–13. 10.1073/pnas.96.12.6908 10359812 PMC22015

[7] Bonten MJ, Slaughter S, Ambergen AW, Hayden MK, van Voorhis J, Nathan C, The role of "colonization pressure" in the spread of vancomycin-resistant enterococci: an important infection control variable. Arch Intern Med. 1998;158:1127–32. 10.1001/archinte.158.10.1127 9605785

[8] Frieden TR, Munsiff SS, Low DE, Willey BM, Williams G, Faur Y, Emergence of vancomycin-resistant enterococci in New York City. Lancet. 1993;342:76–9. 10.1016/0140-6736(93)91285-T 8100912

[9] Boyce JM, Opal SM, Chow JW, Zervos MJ, Potter-Bynoe, Sherman CB, . Outbreak of multidrug-resistant *Enterococcus faecium* with transferable vanB class vancomycin resistance. J Clin Microbiol. 1994;32:1148–53. 8051238 10.1128/jcm.32.5.1148-1153.1994 PMC263627

[10] Henning KJ, Delencastre H, Eagan J, Boone N, Brown A, Chung M, Vancomycin-resistant *Enterococcus faecium* on a pediatric oncology ward: duration of stool shedding and incidence of clinical infection. Pediatr Infect Dis J. 1996;15:848–54. 10.1097/00006454-199610000-00004 8895914

[11] Rubin LG, Tucci V, Cercenado E, Eliopoulos G, Isenberg HD. Vancomycin-resistant *Enterococcus faecium* in hospitalized children. Infect Control Hosp Epidemiol. 1992;13:700–5. 1289397 10.1086/648342

[12] Morris JG Jr, Shay DK, Hebden JN, McCarter RJ Jr, Perdue BE, Jarvis W, Enterococci resistant to multiple antimicrobial agents, including vancomycin. Establishment of endemicity in a university medical center. Ann Intern Med. 1995;123:250–9. 7611590 10.7326/0003-4819-123-4-199508150-00002

[13] Shay DK, Maloney SA, Montecalvo M, Banerjee S, Wormster GP, Arduino MJ, Epidemiology and mortality risk of vancomycin-resistant enterococcal bloodstream infections. J Infect Dis. 1995;172:993–1000. 7561221 10.1093/infdis/172.4.993

[14] Tornieporth NG, Roberts RB, John J, Hafner A, Riley LW. Risk factors associated with vancomycin-resistant *Enterococcus faecium* infection or colonization in 145 matched case patients and control patients. Clin Infect Dis. 1996;23:767–72. 8909842 10.1093/clinids/23.4.767

[15] Karanfill LV, Murphy M, Josephson A, Gaynes R, Mandell L, Hill BC, A cluster of vancomycin-resistant *Enterococcus faecium* in an intensive care unit. Infect Control Hosp Epidemiol. 1992;13:195–200. 1593099 10.1086/646509

[16] Livornese LL, Dias S, Samel C, Romanowskyi B, Taylor S, May P, Hospital-acquired infection with vancomycin-resistant *Enterococcus faecium* transmitted by electronic thermometers. Ann Intern Med. 1992;117:112–6. 1605425 10.7326/0003-4819-117-2-112

[17] Luber AD, Jacobs RA, Jordan M, Guglielmo BJ. Relative importance of oral versus intravenous vancomycin exposure in the development of vancomycin-resistant enterococci. J Infect Dis. 1996;173:1292–3. 8627091 10.1093/infdis/173.5.1292

[18] Bonten MJ, Hayden MK, Nathan C, van Voorhis J, Matushek M, Slaughter S, Epidemiology of colonization of patients and environment with vancomycin-resistant enterococci. Lancet. 1996;348:1615–9. 10.1016/S0140-6736(96)02331-8 8961991

[19] Slaughter S, Hayden MK, Nathan C, Hu TC, Rice T, van Voorhis J, A comparison of the effect of universal use of gloves and gowns with that of glove use alone on acquisition of vancomycin-resistant enterococci in a medical intensive care unit. Ann Intern Med. 1996;125:448–56. 8779456 10.7326/0003-4819-125-6-199609150-00004

[20] Carmeli Y, Samore MH, Huskins WC. The association between vancomycin treatment and hospital-acquired vancomycin-resistant enterococci (VRE): a meta-analysis. Arch Intern Med. 1999;159:2461–8. 10.1001/archinte.159.20.2461 10665895

[21] Ostrowsky BE, Venkataraman L, D'Agata EM, Gold HS, DeGirolami PC, Samore MH. Vancomycin-resistant enterococci in intensive care units: high frequency of stool carriage during a non-outbreak period. Arch Intern Med. 1999;159:1467–72. 10.1001/archinte.159.13.1467 10399898

[22] Samore MH, Lichtenberg D, Saubermann L, Kawachi C, Carmeli Y. A clinical data repository enhances hospital infection control. JAMIA Proceedings of the American Medical Informatics Association Annual Fall Symposium Oct 1997. p. 56–60. PMC2233433 9357588

[23] Murray BE. Vancomycin-resistant enterococcal infections. N Engl J Med. 2000;342:710–21. 10.1056/NEJM200003093421007 10706902

[24] Harris A, Samore M, Carmeli Y. Control group selection is an important but neglected issue in the studies of antibiotic resistance. Ann Intern Med. 2000;133:159. 10896645 10.7326/0003-4819-133-2-200007180-00018

[25] Harris AD, Karchmer TB, Carmeli Y, Samore MH. Methodological principles of case-control studies that analyzed risk factors for antibiotic resistance: a systematic review. Clin Infect Dis. 2001;32:1055–61. 10.1086/319600 11264034

[26] Harbarth S, Cosgrove S, Carmeli Y. Effects of antibiotics on nosocomial epidemiology of vancomycin resistant enterococci (VRE). Antimicrob Agents Chemother. 2002;46:1619–28. 10.1128/AAC.46.6.1619-1628.2002 12019066 PMC127216

[27] Fridkin SK, Edwards JR, Courval JM, Hill H, Tenover FC, Lawton R, The effect of vancomycin and third-generation cephalosporins on prevalence of vancomycin-resistant enterococci in 126 U.S. adult intensive care units. Ann Intern Med. 2001;135:175–83. 11487484 10.7326/0003-4819-135-3-200108070-00009

[28] Harbarth S, Harris AD, Carmeli Y, Samore MH. Parallel analysis of individual and aggregated data on antibiotic exposure and resistance in gram-negative bacilli. Clin Infect Dis. 2001;33:1462–8. 10.1086/322677 11588690

[29] Gerding DN. Is there a relationship between vancomycin-resistant enterococcal infection and *Clostridium difficile* infection? Clin Infect Dis. 1997;25(suppl 2):S206–10. 10.1086/516247 9310680

[30] Donskey CJ, Hanrahan JA, Hutton RA, Rice LB. Effect of parenteral antibiotic administration on persistence of vancomycin-resistant *Enterococcus faecium* in the mouse gastrointestinal tract. J Infect Dis. 1999;180:384–90. 10.1086/314874 10395853

[31] Donskey CJ, Hanrahan JA, Hutton RA, Rice LB. Effect of parenteral antibiotic administration on the establishment of colonization with vancomycin-resistant *Enterococcus faecium* in the mouse gastrointestinal tract. J Infect Dis. 2000;181:1830–3. 10.1086/315428 10823795

[32] Donskey CJ, Chowdhry TK, Hecker MT, Hoyen CK, Hanarahan JA, Hujer AM, Effect of antibiotic therapy on the density of vancomycin-resistant enterococci in the stool of colonized patients. N Engl J Med. 2000;343:1925–32. 10.1056/NEJM200012283432604 11136263 PMC4370337

